# Dreaming of Better Treatments: Advances in Drug Development for Sleep Medicine and Chronotherapy

**DOI:** 10.1111/jsr.70087

**Published:** 2025-05-10

**Authors:** Brooke A. Prakash, Ishani Shah, Guohao Ni, Sridhar Vasudevan, Aarti Jagannath, Russell G. Foster

**Affiliations:** ^1^ Department of Pharmacology University of Oxford Oxford UK; ^2^ Nuffield Department of Clinical Neurosciences, Sleep and Circadian Neuroscience Institute (SCNi) University of Oxford Oxford UK

**Keywords:** chronotherapy, circadian, GABA, glymphatic clearance, kinases, melatonin, neurodegeneration, orexin, sleep, sleep medicine

## Abstract

Throughout history, the development of new sleep medicines has been driven by progress in our understanding of the mechanisms underlying sleep. Ancient civilisations used their understanding of the sedative nature of natural herbs and compounds to induce sleep. The discovery of barbiturates and bromides heralded a new era of synthetic sleep medicine in the 19th century. This was followed by the development of benzodiazepines that were used to inhibit signalling throughout the brain by promoting gamma‐amino butyric acid release and thereby produce loss of consciousness. As our understanding of sleep has deepened, newer therapies have more specifically targeted the wake‐inducing neurotransmitter orexin with fewer side effects. Given the newly highlighted role of kinases in sleep/wake regulation, we predict that the next breakthroughs in sleep medicine will likely target these kinases. Given the fundamental role that sleep plays in maintaining brain health through processes such as glymphatic clearance, sleep medicine has therapeutic potential beyond just sleep. Recent evidence suggests that sleep disruptions directly contribute to the build‐up of pathological neuronal proteins in neurodegenerative disorders. Therefore, sleep medicine could improve prognosis in disorders such as these. Great attention must be paid to the mechanism of action of each sleep medicine, however, as sleep medicines which do not fully mimic sleep could actually worsen disease progression.

## 1 The History of Sleep Medicine

Sleep disorders have been a global concern since ancient times, and treatments have evolved with each civilisation's understanding of sleep. Records are available from Ancient Egypt regarding the development of various herbal potions and remedies for treating sleep disorders (Zaki and Morsy 2023). The Romans had the cultural belief that sleep disturbances are due to nightmares caused by visiting spirits (Bonnema and Morgenthaler 2010). The Greeks took a more scientific approach, with Hippocrates reporting the observation that age and certain medical factors, namely exercise, may affect sleep. Based upon his findings, Hippocrates pioneered the idea of treatments for sleep that were specifically tailored to each patient based on their pathology (Nomikos et al. 2016). Some of the oldest forms of treatment were generally botanical, with various herbs and plants being used as sleep inducers (Thorpy 2011). Of these older treatments, opium, alcohol and hypnotic herbal medicines gained popularity for inducing sleep. Some of these herbal medicines have stood the test of time, such as valerian root, which is still utilised today in traditional medicine for treating sleep‐related disorders like insomnia (Thorpy 2011). To modern society, these ancient practices may appear naive, but they nonetheless represent humankind's first efforts to understand sleep disorders.

The 19th century was a revolutionary period for the treatment of sleep disorders, where new pharmacological interventions were introduced. Morphine was isolated in 1805 and soon became widely used as a sleep aid, and it was not until the middle of the 19th century that its addictive properties were realised (Abraham and Sheppard 2014). The 20th century saw the rise of the first synthetic sleep medications such as chloral hydrate, bromides and barbiturates, which introduced sedative‐hypnotic medication into clinical practice (Johns 1975; Mendelson 2012). After their introduction, barbiturates rapidly became the first choice for treating sleep disorders. However, excessive use was soon linked to safety concerns regarding addiction. Indeed, the abuse of sleeping pills presented a significant health issue throughout the mid‐19th and most of the 20th centuries (Shiroma and Kramer 2008; Aebischer and Rieder 2020).

## 2 Mid‐20th Century Breakthrough: Circuit‐Based Pharmacological Approaches

**FIGURE 1:**
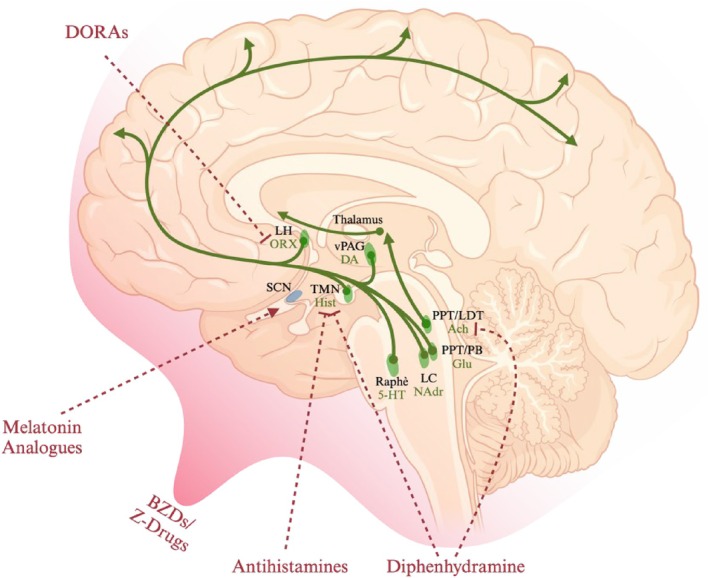
Summary of neuronal circuits in sleep/wake and the drugs that act on them. The ascending arousal system regulates sleep/wake and alertness via projections from the brainstem and posterior hypothalamus to the forebrain, which is interconnected with the cortex. Sleep/wake state is regulated by wake‐promoting and sleep‐promoting neural circuits which switch states through mutual inhibition. Wake‐promoting neurons in the ARAS are made up of dopaminergic, orexinergic, noradrenergic, serotonergic, histaminergic, cholinergic and glutamatergic nuclei which interact via excitatory neurotransmitters to promote arousal and inhibit sleep‐promoting neurons. Inhibition of these interactions by inhibitory neurotransmitters (primarily GABA) promotes sleep. BZDs and Z‐Drugs potentiate the inhibitory effects of GABA receptors throughout the brain to produce loss of consciousness. Antihistamines promote sleep by inhibiting the action of wake‐promoting histamine. Similarly DORAs inhibit orexinergic signalling and diphenhydramine inhibits both histaminergic and cholinergic signalling. Melatonin analogues bind to melatonin receptors in the SCN to exert a very small sleep‐promoting effect. Abbreviations not in main text: locus coeruleus (LC); tuberomammillary nucleus (TMN); laterodorsal tegmental (LDT); pedunculopontine nuclei (PPT); Neurotransmitters: glutamate (Glu); orexin (ORX); acetylcholine (ACh); dopamine (DA), serotonin (5‐HT); histamine (His); noradrenaline (NA); lateral hypothalamus (LH); parabrachial nucleus (PB). Created in BioRender. Prakash, BA. (2025) https://BioRender.com/w74y872.

The next advance in sleep medicine coincided with growing research into the neuronal control of sleep/wake regulation. The first hypothesis emerged during World War I when neurologist Baron Constantin von Economo studied patients with Encephalitis Lethargica (Dickman 2001). He proposed the existence of a ‘sleep centre’ in the anterior hypothalamic region which controlled ‘brain sleep’ by inhibiting thalamic and cerebral cortical activity (Economo 1930). Subsequent research, notably by Ranson, Moruzzi and Magoun and Saper, developed and refined this hypothesis into a circuit‐based model of sleep–wake regulation (Moruzzi and Magoun 1949; Ranson 1939; Lu et al. 2006; Saper and Fuller 2017; Saper et al. 2010). This model describes a dynamic antagonism between sub‐cortical sleep‐promoting and wake‐promoting circuits mediated by fast neurotransmitters like gamma‐amino butyric acid (GABA) and orexin. These circuits function as switches, with no single neurotransmitter being essential for the transition between sleep and wake states. This ascending arousal system, also known as the ascending reticular activating system (ARAS), regulates sleep/wake and alertness via projections from the brainstem and posterior hypothalamus to the forebrain, which is interconnected with the cortex (Figure 1). Sleep/wake state is regulated by wake‐promoting and sleep‐promoting neural circuits which switch states through mutual inhibition. Wake‐promoting neurons in the ARAS are made up of dopaminergic, orexinergic, noradrenergic, serotonergic, histaminergic, cholinergic and glutamatergic nuclei which interact via excitatory neurotransmitters to promote arousal and inhibit sleep‐promoting neurons (Eban‐Rothschild et al. 2018; Scammell et al. 2017; De Luca et al. 2022). Inhibition of these interactions by inhibitory neurotransmitters (primarily GABA) promotes sleep. Sleep‐promoting neurons in the ventrolateral preoptic nucleus (VLPO) and median preoptic nucleus (MnPO) send GABAergic projections to most components of ARAS to inhibit arousal (Saper et al. 2010; Saper et al. 2001).

Shortly after von Economo's foray into sleep research, the first anti‐histamines were discovered in the 1930s. While they were developed for treating allergic disorders, a side effect of ‘drowsiness’ was quickly observed (Friedlaender and Feinberg 1946). These first‐generation antihistamines were blood–brain barrier permeant and thus could interfere with the histaminergic neurons involved in promoting wakefulness (Roth et al. 1987). Second‐generation antihistamines that did not cross the blood–brain barrier were developed explicitly to reduce the risk of sedation (Slater et al. 1999). However, first‐generation antihistamines are widely available over the counter (OTC) as allergy medications and are the most used OTC treatment for chronic insomnia in the US. The National Institutes of Health cited significant concern about the lack of evidence to support the use of antihistamines to promote sleep given the serious adverse effects of daytime sedation and reduced cognitive function (National Institutes of Health 2005).

As with antihistamines, benzodiazepines (BZDs) were discovered based upon their observed sedative effects, with a limited understanding of their mechanism of action by Leo Sternbach in 1955 (Junkes et al. 2024). The first BZDs, chlordiazepoxide and diazepam (Valium), were approved in the early 1960s for anaesthesia and the treatment of emergency seizures and muscle spasms (Riss et al. 2008; McClish 1966). Although not initially approved for insomnia, they were commonly prescribed for severe insomnia and became the most prescribed drugs in the world by the 1970s for anxiety, depression, insomnia and stress (Ashton 2005). While less toxic in overdose than barbiturates, BZDs carried risks of tolerance, dependence and addiction. These drawbacks were initially overshadowed by their popularity and the enthusiasm of clinicians to prescribe them. However, over time their misuse became evident, with BZDs frequently implicated in drug‐related overdoses, often co‐abused with alcohol and opioids (Substance Abuse Mental Health Services Administration 2011).

A significant breakthrough came in 1977 when Mohler and Okada identified the gamma‐amino butyric acid (GABA)‐alpha receptor as a target for diazepam (Mohler and Okada 1977a, 1977b; Braestrup and Squires 1977; Braestrup et al. 1977). That same year, Macdonald and Barker demonstrated that barbiturates also target the GABA receptor at a different site (Macdonald and Barker 1978). GABA is one of the most abundant inhibitory neurotransmitters, and these discoveries emphasised the fundamental role of neurotransmitter GABA modulation in sleep regulation (Sears and Hewett 2021; de La Ochoa‐Paz et al. 2021). BZDs are CNS depressants that exert their sedative effect by potentiating the inhibitory action of GABA through allosterically binding to GABA‐A receptors, reducing the output of excitatory neurotransmitters in sleep‐promoting nuclei such as the VLPO and MnPO, which project to most wake‐promoting nuclei. Therefore, BZDs inhibit neuron firing across the arousal circuit, resulting in a hypnotic and sedative effect (Charney et al. 2001).

While BZDs cause rapid sedation, the widespread distribution of GABAergic neurons in the brain and the lack of receptor specificity lead to broad neuronal inhibition. This increases the likelihood of side effects such as daytime drowsiness, cognitive impairment and disruptions to natural sleep architecture (Manconi et al. 2017). These limitations underscore the challenges inherent in neurotransmitter‐based strategies. Despite this, BZDs continue to be the most widely used treatment for chronic insomnia, often prescribed for durations exceeding the recommended guidelines in Europe (Soyka et al. 2023).

In the late 1980s and early 1990s, Z‐drugs, including zolpidem (marketed as Ambien), zopiclone and zaleplon, were introduced to the market and promoted as safer alternatives to classical BZDs. This perception contributed to zolpidem becoming the global market leader in hypnotics for over two decades following FDA approval in 1992 (U.S. Food and Drug Administration 2005). Their shorter half‐life was initially believed to reduce the risk of dependence and minimise residual daytime carry‐over (Schifano et al. 2019). However, subsequent evaluations suggest that these claims were not adequately supported by clinical evidence.

Despite their structural differences, Z‐drugs exert their effects by binding to the same GABA‐A1 receptor complex as BZDs, leading to similar pharmacological profiles, including sedation, tolerance, dependence and withdrawal symptoms (Wilson et al. 2019). Moreover, Z‐drugs have been associated with complex sleep‐related behaviours such as sleepwalking, sleep‐driving and other parasomnias, prompting significant public concern (Hoque and Chesson Jr. 2009). These adverse effects culminated in a class‐action lawsuit against Sanofi, the manufacturer of Ambien, and the coining of the term ‘Ambien Zombies’ in popular media (Pirestani 2017). Nevertheless, the enduring influence of BZDs and Z‐drugs on the field is evidenced by the continued research into safer, more selective drugs that target the GABA‐A receptor, such as Dimdazenil, approved in China in 2023 to treat insomnia (Syed 2024; Huang et al. 2024; Wang et al. 2023). This highlights the impact neurotransmitter‐targeted approaches in sleep medication research, and the prevailing paradigm of targeting the arousal systems to induce sleep.

## 3 Dual Orexin Receptor Antagonists: Targeted Neurotransmitter Modulation

The discovery of orexin/hypocretin in 1998 marked a new era of sleep–wake regulation research (de Lecea 2012; Sakurai et al. 1998; Lin et al. 1999; Chemelli et al. 1999). It was found that dogs inherit narcolepsy (a disorder characterised by increased daytime sleepiness and the inability to control sleep/wake state) through a mutation in the hypocretin (orexin) receptor 2 gene (*Hcrtr2*) (Lin et al. 1999). This phenotype was reproduced in *orexin* knockout mice, and the findings were eventually extended to humans, where it was found that narcoleptic patients lose up to 95% of orexinergic neurons (Chemelli et al. 1999; Thannickal et al. 2000; Peyron et al. 2000). Orexin was therefore recognised as a key player in stabilising wakefulness by inhibiting sleep‐promoting neurons. The contrapositive idea that overactivity of orexinergic neurons underlies human cases of insomnia has been more difficult to prove. Mice that overexpress prepro‐orexin show increased sleep fragmentation and reduced rapid eye movement (REM) sleep (Willie et al. 2011). In addition, a study of human patients found elevated plasma levels of orexin‐A that correlated with insomnia severity. However, the researchers were unable to identify a genetic link to the orexin pathway in insomnia patients and did not establish a central role for orexin, as it was only detected peripherally (Tang et al. 2017).

Nevertheless, the undisputed role of orexin in wakefulness led to the development of dual orexin receptor antagonists (DORAs), a new class of sedatives which includes FDA‐approved Suvorexant, Lemborexant and Daridorexant (in Europe and UK too) for the treatment of insomnia (Merck Sharp and Dohme Corp 2014; Idorsia Pharmaceuticals US Inc 2022; Eisai R&D Management Co. Ltd 2019). DORAs represent a more targeted approach by selectively inhibiting the wake‐promoting pathway regulated by the neurotransmitter orexin. DORAs selectively bind to and inactivate orexin receptors 1 and 2, suppressing the release of histamine in the lateral hypothalamus, hippocampus and prefrontal cortex to inactivate wakefulness (De Luca et al. 2022). Preclinical literature has suggested that this selectivity could preserve sleep architecture and minimise side effects like cognitive impairment and dependency (Roch et al. 2021). Clinical trials of DORAs have shown improvements in sleep onset and maintenance in patients with insomnia, although further research is needed to determine whether DORAs replicate natural sleep (Snyder et al. 2016; Di Marco et al. 2024). Emerging orexin receptor‐targeting drugs are undergoing clinical trials for treatments for disorders with insomnia symptoms, such as ORX750 and ALKS 2680 for narcolepsy and seltorexant for major depressive disorder, demonstrating the promise of this selective approach (Ciccone 2023; Yee et al. 2023; Recourt et al. 2019).

Although DORAs are not associated with misuse, as relatively new drugs, DORAs require long‐term evaluation to fully assess their safety and efficacy. After all, it took over a decade for clinicians and researchers to fully recognise the significant side effects associated with BZDs. DORAs face other challenges such as higher costs and limited availability, which may restrict access. Nevertheless, their targeted mechanism offers hope for a safer and more effective class of sleep medication than traditional sedatives.

## 4 Melatonin Analogues: Mimicking the ‘Sleep Hormone’

Melatonin is often referred to as a ‘sleep hormone’, though this is inaccurate. In mammals, melatonin is made mainly in the pineal gland, but it is also produced in the eyes where it probably plays an important role in eye health (Felder‐Schmittbuhl et al. 2024; Foster 2021). For sleep regulation, the pineal gland is most relevant as it is regulated by the ‘master circadian pacemaker’ located in the suprachiasmatic nuclei (SCN). The SCN drives a pattern of melatonin release, where levels rise at dusk, peak in the blood around 02.00–04.00 am and then decline around dawn. Bright light, detected by the retina, also acts to inhibit melatonin production. The net result is that melatonin acts as a biological marker of dark exposure (Arendt 2005). While melatonin production occurs at night during sleep in day‐active animals such as humans, nocturnal animals like mice and rats also produce melatonin at night when they are active—thus debunking the universal ‘sleep hormone’ rhetoric. In humans, individuals who have lost their neural connection to the pineal and do not produce melatonin (e.g., tetraplegic individuals and people on beta‐blockers) still have sleep/wake rhythms and show only small changes in their sleep patterns (Foster 2021). Melatonin is detected by receptors in the SCN; thus the nighttime release of pineal melatonin provides a signal to tell the SCN how long it has been dark, reinforcing the light signals from the eyes. There are studies suggesting that melatonin may help reduce the time it takes to fall asleep (sleep latency) and increase total sleep time. However, the demonstrated effect is small, shortening the time to fall asleep by around 10–20 min at best (Arendt and Skene 2005). In summary, light‐regulated melatonin seems to be a mild modulator, rather than a driver, of sleep. Nevertheless, melatonin analogues have been developed in an attempt to regulate sleep.

Melatonin analogues are synthetic compounds that mimic the action of melatonin by targeting melatonin receptors MT1 and MT2 in the brain. These compounds have been prescribed in disorders of circadian regulation including transient insomnia, delayed sleep‐wake phase disorder and non‐24 h sleep‐wake disorder.

One of the first melatonin analogues developed clinically is the MT1/2 selective agonist Ramelteon, which was approved as a non‐sedative hypnotic for the treatment of insomnia as it reduces sleep latency (Erman et al. 2006). While it has several advantages including reduced dependency and lack of rebound insomnia and cognitive/psychomotor effects which are typically seen with sedative hypnotics, it shows relatively modest efficacy when compared with traditional hypnotics (Pandi‐Perumal et al. 2009).

Tasimelteon, marketed as Hetlioz, was the first approved treatment for Non‐24‐Hour Sleep–Wake Disorder (Non‐24). In this condition where no light input reaches the circadian clock, an alternative entraining stimulus is required. The SCN expresses MT1/MT2 receptors, and thus Tasimelteon was proposed as an alternative zeitgeber. A randomised placebo‐controlled clinical trial showed that once daily treatment over 26 weeks, and continuous treatment beyond, maintained entrainment as evidenced by dim light melatonin onset measurements. However, entrainment rates were limited (20%–35% vs. 5% placebo) (Lockley et al. 2015).

Agomelatine is an MT1 and MT2 agonist with additional 5‐HT2C antagonist activity. As such, it is approved for major depressive disorder (MDD) with comorbid sleep disturbances (Taylor et al. 2014). Like melatonin, it reduces sleep latency and simultaneously improves circadian entrainment and allied physiology including mood (Fornaro et al. 2010). Seltorexant, a dual MT receptor agonist and orexin receptor antagonist, is currently under development for depression‐related sleep disturbances (Mesens et al. 2024).

Thus melatonin analogues overall offer an alternative to traditional sedatives, particularly for circadian rhythm‐related sleep disorders. Their advantages include minimal risk of dependence, fewer cognitive side effects and better regulation of natural sleep processes. However, limited efficacy has thwarted wide clinical application.

## 5 Adverse Side Effects: Addiction and Dependency

Despite differing mechanisms of action, many sleep medications have been plagued with problems of addiction and dependence. Marilyn Monroe and Elvis Presley are just a couple of famous examples of people who have died from sedative overdose (Pagel and Parnes 2001). Many hypnotics, such as barbiturates, have anxiolytic and relaxing effects that can lead to abuse by those seeking to change their mood and avoid stress and/or pain (Nattala et al. 2011). Indeed, sleep medications fundamentally alter neurochemistry, and chronic use can lead to adaptations in the brain. These adaptations increase drug tolerance, necessitating a higher dose and thus leading to a self‐perpetuating cycle in which patients become dependent upon medication to sleep, and discontinuation can lead to serious withdrawal symptoms. For this reason, it is recommended that the majority of sleep aids be taken on a short‐term (< 4 weeks) basis and only approved for long‐term use in extreme cases (Riemann et al. 2023).

## 6 Our Modern Understanding of the Mechanisms Underlying Sleep: The Two Process Model

**FIGURE 2:**
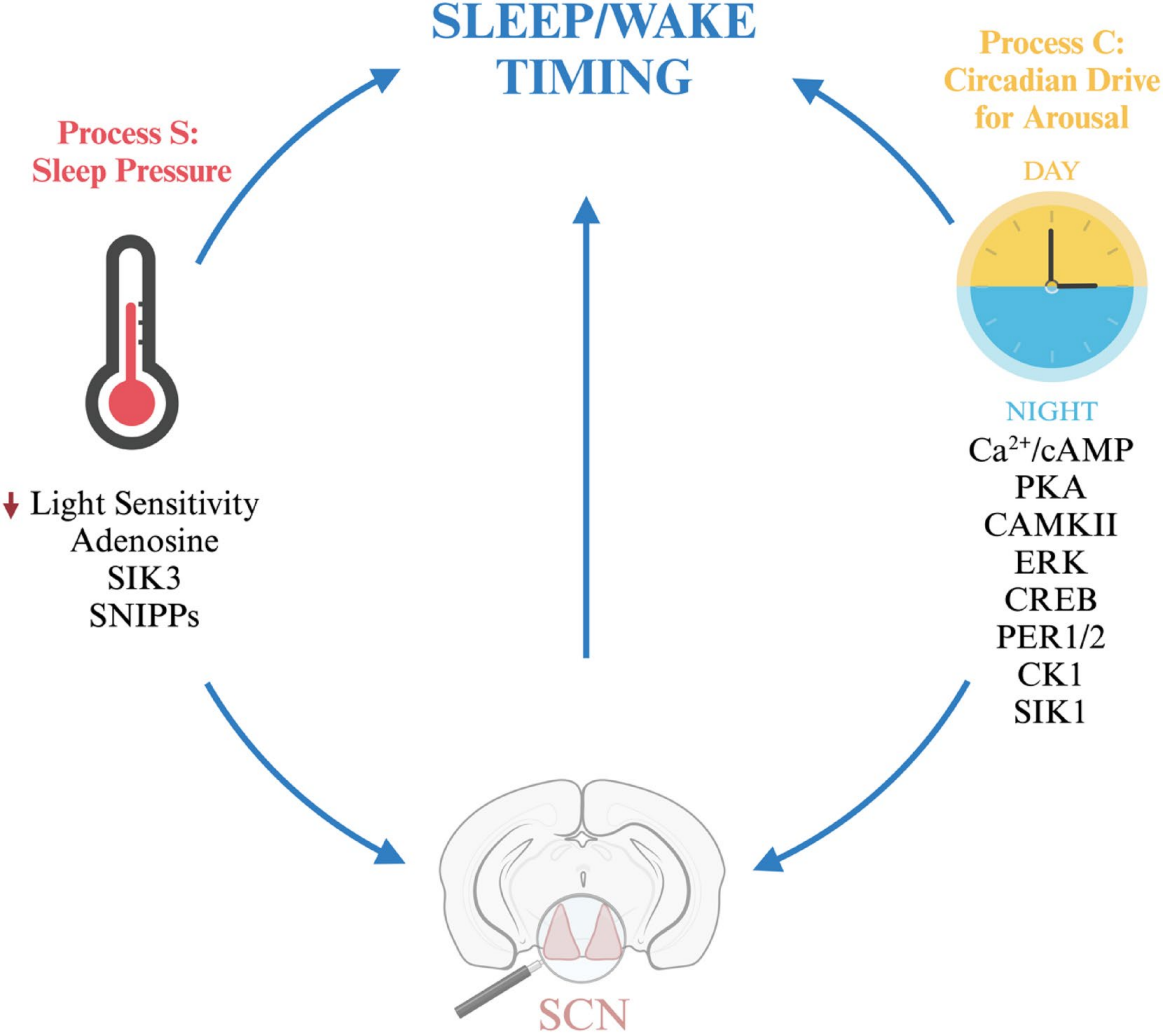
Molecular determinants of interactions within the two process model. Sleep timing is determined by process C (the circadian drive for arousal) and process S (sleep pressure). Process C is timed with the external light/dark cycle. Light enters the eye, stimulating the release of Ca^2+^ and cAMP in the SCN, which activate PKA, CAMKII and ERK pathways. Elevated cAMP levels lead to the phosphorylation of CREB, which induces PER transcription, setting the circadian clock to the light cycle. The kinase CK1 controls the stability of PER through phosphorylation, thus playing a key role in circadian timing. Light‐induced PKA activity also increases SIK transcription. SIK1 then blunts the effect of light on the clock, limiting how quickly the clock can readjust to light exposure. Adenosine is another player in circadian timing, which is activated by elevated Ca^2+^ and alters PER levels through the ERK pathway to inhibit the effect of light on the clock. Process S is driven by the accumulation of adenosine, SIK3 and the SNIPPs, which accrue with wake duration. See text for abbreviations. Created in BioRender. Prakash, B.A. (2025) https://BioRender.com/w34w313.

At the heart of the challenge of developing new drugs in sleep medicine is the fact that we still do not fully understand sleep as a process. As such, it is difficult to design drugs to mimic a process of which we do not know the mechanism or purpose. Over 40 years ago, two‐process model of sleep regulation was first proposed, and recent advances in sleep research have elucidated some of the molecular mechanisms underpinning it (Borbely 1982). As suggested in the name, this model of sleep describes two processes, S and C, which coordinate to determine sleep timing and sleep duration (Figure 2). Process S is a manifestation of sleep need (pressure)—it declines during sleep and increases during wakefulness. Process C occurs independently of sleep and is determined by the circadian drive for arousal. It therefore oscillates in a daily cycle. Process C is driven by a well characterised cell autonomous molecular clock aligned to the environmental light–dark cycle by a master clock in the SCN (Ashton et al. 2022). At its core, the clock consists of transcriptional drivers CLOCK AND BMAL1, which drive the transcription of multiple clock‐controlled genes, including their repressors, PERIOD1/2 and CRYPTOCHROME. This negative feedback loop takes 24 h to complete and thus regulates the tissue‐specific oscillation of the transcriptome in synchrony with the external light/dark cycle.

According to the two process model, sleep onset occurs when sleep pressure is high and the circadian drive for arousal is low (typically at the end of the wake phase). Conversely, wake occurs when sleep pressure has dissipated below a critical threshold and the circadian drive for arousal is high (typically at the end of the sleep phase). The model was proposed based on observations of electroencephalogram (EEG) data measuring slow wave sleep (SWS) in rats. SWS is viewed as a correlate of process S and increases after sleep deprivation. The rats were subjected to 24 h of sleep deprivation starting at the onset of their wake period. During recovery sleep, an initial increase in SWS occurred in the first 3 h of the dark phase, followed by another rise 12 h later in the first 3 h of light onset—suggesting process S drove the first increase and process C drove the second (Borbely and Neuhaus 1979).

Understanding the molecular pathways by which Process S and C coordinate to regulate sleep/wake timing can give rise to new mechanistic targets for the development of novel therapeutics. While any common molecular substrates of Process S and C remain to be conclusively identified, recent developments in kinase‐related targets as well as adenosine signalling have shown promise, as summarised below.

## 7 Looking Forward: The Development of New Sleep Medicines

It seems likely that the development of new sleep medicines will be based upon an increased understanding of the mechanisms involved in sleep generation and regulation. Although sleep generation has been represented as process S and C, it is clear that there are multiple pathways that contribute to each of these drivers. One such mechanism that has recently been highlighted is protein phosphorylation. Wang et al. demonstrated that the mouse brain proteome becomes progressively phosphorylated during sleep deprivation and this phosphorylation dissipates upon recovery sleep. Indeed, by comparing the phosphorylated brain proteins between mice that were sleep deprived for 6 h and the *Sleepy* mutant mouse model (Sik3^Slp/+^) which has constitutionally high sleep need, the researchers identified 80 proteins whose phosphorylation state increases with sleep need across both models (Wang et al. 2018). These findings suggested that phosphorylation of these Sleep‐Need‐Index‐PhosphoProteins (SNIPPs) could be an important physiological correlate of process S.

Brüning et al., meanwhile, have also tied phosphorylation to process C. They found that 30% of synaptic phosphoproteins in the mouse brain are phosphorylated in a circadian fashion, with the phosphorylation of one group peaking at dawn and the other at dusk. Sleep deprivation prevented the phosphorylation of all but 41 of these phosphopeptides, suggesting a mechanism for how sleep deprivation can ‘overpower’ the circadian clock (Bruning et al. 2019). These findings are in line with previous evidence, which shows that kinases are directly involved with how light regulates the timing of process C. Light directly entrains the circadian clock by stimulating an increase of intracellular Ca^2+^ and cAMP levels in the SCN, which in turn activate kinase signalling pathways, including the protein kinase A (PKA) and Ca^2+^/calmodulin‐dependent protein kinase II (CaMKII) pathways. The primary mechanism of photic entrainment is believed to be regulated by the increased cAMP levels that activate the transcription factor cAMP response element‐binding protein (CREB), which in turn increases transcription of the key circadian gene *Per1/2* (Ashton et al. 2022).

As kinases are responsible for driving protein phosphorylation, we propose that drugs targeting kinase activity will provide a new therapeutic avenue for the regulation of sleep. Indeed, since 2001, over 70 kinase inhibitors have been approved by the FDA, predominantly for cancer treatment (Cohen et al. 2021). Many of these kinase inhibitors are blood–brain barrier permeant, making them promising candidates for treating sleep. Kinase inhibitors are not without their challenges, however. Many approved protein kinase inhibitors are not specific for their target and exhibit polypharmacology, which can lead to side effects (Cohen et al. 2021). In addition, even if these drugs were specific, kinases themselves are often involved in multiple pathways and thus whole‐body inhibition or activation is likely not an option. More research is required, therefore, to identify which kinases drive sleep processes and then design molecules that alter their activity in sleep pathways without deleterious side effects.

Salt‐inducible kinases (SIKs) represent a potential therapeutic target for sleep medicine. The *Sleepy* mouse model mentioned previously was generated through deletion of exon 13 in the *Sik3* gene, resulting in a gain‐of‐function SIK3 variant which has constitutively high sleep pressure as demonstrated by increased SWS (Funato et al. 2016). Mutation of a PKA phosphorylation site in *Sik3* phenocopies this amplified sleep need (Honda et al. 2018). Furthermore, mouse models with mutations in PKA binding sites that prevent the nuclear shuttling of SIK1‐2 also exhibit higher sleep pressure (Park et al. 2020). Thus, the PKA‐SIK axis appears to be crucial in the regulation of process S. We, meanwhile, have tied SIK1 activity to process C, showing that light induces *Sik1* expression in the master circadian clock and this expression acts as a ‘brake’ on entrainment to a new light/dark cycle—suppressing the phase‐shifting effect of light on the circadian clock (Jagannath et al. 2013). Furthermore, light‐induced SIK1 activity is independent of sleep pressure (Taylor et al. 2021). Therefore, the SIK family represents another promising node connecting process S and process C.

Adenosine signalling has also received interest as a potential therapeutic pathway that regulates process S and C. Using transgenic mice which overexpress adenosine kinase (a kinase that promotes the metabolic clearance of adenosine), we demonstrated that the accumulation of adenosine is necessary for the build‐up of SWS that occurs after sleep deprivation. Thus, adenosine is a physiological correlate for process S. However, it also feeds into process C, as adenosine inhibits the pathways that are activated by light in the brain, thereby directly altering the expression of circadian clock genes (Jagannath et al. 2021). This work has raised an interest in the therapeutic use of adenosine receptor antagonists to synchronise circadian rhythms and thus sleep to the appropriate time of day (Jagannath et al. 2022; Vasudevan et al. 2019). The signalling underlying adenosine's sleep‐modulating capabilities is also kinase‐dependent. Adenosine antagonists increase cytosolic Ca^2+^, which increases the phosphorylation of the kinase ERK1/2, thereby activating it (Jagannath et al. 2021). Pharmacological inhibition of ERK phosphorylation and genetic deletion of *Erk* have both been shown to decrease sleep and increase wakefulness in mice (Mikhail et al. 2017). In the SCN where adenosine receptors are mainly inhibitory, adenosine antagonists inhibit the activation of PKA. This increases cAMP levels and ultimately increases phosphorylation of CREB, which increases transcription of the key circadian gene *Per1/2* (Jagannath et al. 2021).

Indeed, many kinases control the expression of *Per* to alter sleep and circadian timing. Of all the core clock proteins, only PER's rhythmic expression is crucial for the generation of circadian rhythmicity (Chen et al. 2009). Nuclear entry of PER is necessary for it to exert its negative regulation of circadian gene expression. Its nuclear translocation is facilitated through phosphorylation by casein kinase I ɛ/δ (CK1ɛ/δ), though this phosphorylation also reduces PER stability (Keesler et al. 2000; Vielhaber et al. 2000; Lee et al. 2001). CK1‐dependent phosphorylation of PER plays a crucial role in determining sleep timing in humans, as a mutation in the phosphorylation site was identified in patients with familial advanced sleep phase syndrome (Toh et al. 2001; Xu et al. 2005; Shanware et al. 2011). Accordingly, mouse studies have found that CK1δ inhibitors can be used to entrain arrhythmic mice to their light–dark cycle (Meng et al. 2010). Thus, CK1δ inhibitors could present another therapeutic option in targeting process C.

An increase in cytosolic Ca^2+^ alters sleep processes not only through ERK, but also through Ca^2+^/calmodulin‐dependent protein kinase II (CaMKII)β. Entry of Ca^2+^ through NMDA receptors and voltage‐gated Ca^2+^ (Ca_v_) channels hyperpolarizes neurons, promoting longer sleep duration and SWS. CAMKII is a known modulator of Ca_v_ channels; therefore, it is perhaps unsurprising that genetic *Camk2a* and *Camk2b* knockout mice exhibit reduced sleep duration (Tatsuki et al. 2016). Modulation of CaMKIIβ activity alters sleep duration, with specific sites being linked to the induction and maintenance of sleep in mice (Tone et al. 2022). CaMKIIβ therefore also presents therapeutic potential for sleep. It is worth noting, however, that most of these studies connecting the phosphoproteome to sleep have been conducted in mice, so further validation is required in humans.

## 8 Alternative Inputs to Sleep Regulation

Beyond our traditional understanding of neurotransmitter systems and their influences on sleep regulation, there has been recent interest in alternative inputs into sleep regulation. Specifically, two important studies have identified different molecular mechanisms that helped to reshape our understanding of sleep regulation: the role of antimicrobial peptides in immune‐sleep interactions and the role of dietary protein sensing in the gut‐brain signalling axis.

A group of researchers discovered that an antimicrobial peptide called Nemuri in *Drosophila* is capable of modulating sleep directly, serving both a role in immune defence and promoting sleep (Toda et al. 2019). The idea originated from the observation that Nemuri expression increases during both infection and sleep deprivation, especially in a key sleep‐promoting region of the fly brain called the dorsal fan‐shaped body. The observation demonstrated a bidirectional link between immune function and sleep homeostasis. Interestingly, Nemuri mutants show impaired sleep recovery after sleep deprivation and reduced infection‐induced sleep. This relationship emphasises an evolutionary adaptation of Nemuri where immune defence and sleep are mechanistically linked and integrated, potentially explaining why sleep increases during illness across different species.

Furthermore, in relation to this immune‐sleep axis, another group of researchers discovered that a gut‐secreted peptide typically responsive to dietary protein intake, called CCHa1, influences both sleep quality and depth of sleep (Titos et al. 2023). CCHa1 is produced by enteroendocrine cells and increases specifically with the consumption of protein and not fat or sugar intake. It ultimately affects sleep through dopaminergic neurons. This gut‐brain axis seems to be evolutionarily conserved as protein‐rich diets improve sleep quality in mice, lengthening sleep episodes and increasing REM sleep. These experimental findings suggest the gut‐brain axis is an additional potential therapeutic target for sleep intervention.

These two discoveries underline the fact that sleep is both dynamic and a whole‐body phenomenon that integrates information from immune responses, peripheral gut signalling and metabolic status. The discovery of these alternative pathways highlights the potential for new therapeutic targets that extend beyond traditional neurotransmitter‐based methods. Sleep regulation is a far more complex process than is currently depicted, involving multiple organ systems that work in combination to maintain our sleep homeostasis. The existence of molecules like Nemuri and CCHa1 suggests that throughout evolution, complicated mechanisms have arisen to integrate numerous physiological processes with sleep regulation, ensuring that sleep serves multiple biological functions simultaneously (Foster 2018).

## 9 Sleep Medicine: Implications Beyond Sleep in Neurodegeneration

The development of drugs for sleep and circadian disruptions has potential beyond just the treatment of sleep. Given that sleep is a complex process arising from the interaction of multiple neurotransmitter pathways and brain structures, as previously described, it can be viewed as a readout of general brain health. Indeed, sleep and circadian disruptions (SCRDs) are often comorbidities, presenting alongside other diseases. Multiple single nucleotide polymorphisms in core clock genes have been associated with neuropsychiatric and neurodegenerative diseases (see table 1 in Jagannath et al. 2017). However, it is currently an open question whether the SCRDs are symptoms of these diseases or if they actually contribute to disease progression. An ever‐growing body of evidence now supports the latter (Foster 2020).

**FIGURE 3:**
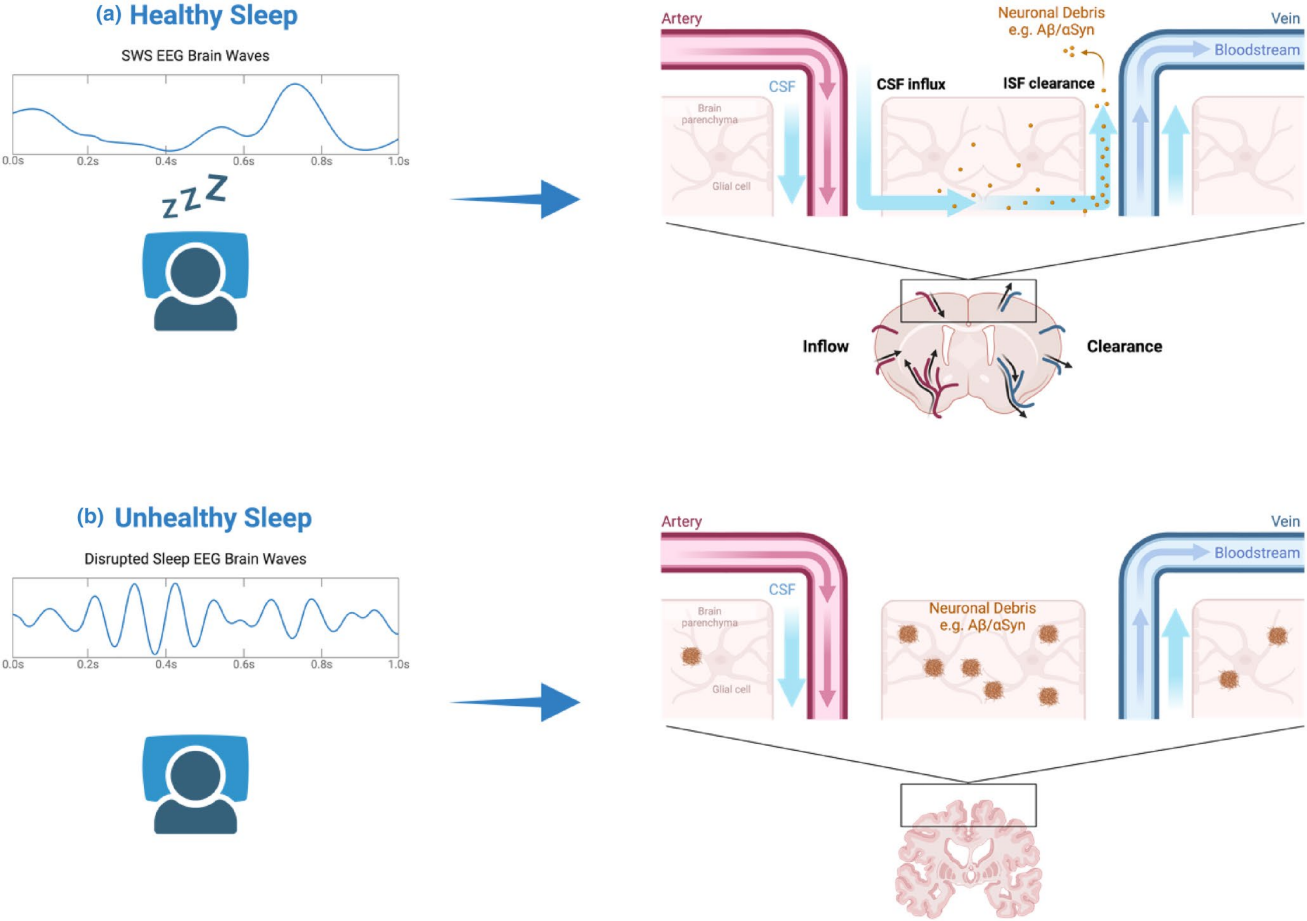
The role of sleep in brain health. (A) Under healthy sleeping conditions, glymphatic clearance occurs during SWS. CSF flows through the perivascular spaces of the arteries into the brain and mixes with interstitial fluid (ISF) where it picks up neuronal debris. It then effluxes out of the brain. (B) This process is a fundamental aspect of sleep that is disrupted in many neurodegenerative disorders where SWS is reduced or fragmented. The build‐up of pathological proteins drives neuronal death. Despite producing loss of consciousness, BZDs and Z‐drugs reduce SWS and therefore reduce glymphatic clearance. See text for abbreviations. Created in BioRender. Prakash, BA. (2025) https://BioRender.com/p47f020. Adapted from ‘Interstitial Solute & Fluid Clearance in the Glymphatic System’ retrieved from https://app.biorender.com/biorender‐templates.

SCRDs are an early key symptom in humans for many neurodegenerative diseases, including Alzheimer's Disease (AD) and Parkinson's Disease (PD). In most cases, SCRDs appear first, up to decades before any of the characteristic symptoms (Musiek and Holtzman 2016). A hallmark of AD is the build‐up of amyloid beta (Aβ) plaques in the brain. These plaques continually aggregate along with tau protein, causing neuronal death and thus neurodegeneration. In PD, neuronal death is driven by the accumulation of misfolded α‐synuclein (αSyn). It has been suggested that SWS is critical in these disorders for removing the pathogenic proteins through glymphatic clearance (Figure 3) (Ju et al. 2017; Morawska et al. 2021). Given that PD and AD patients exhibit progressively less SWS, it is possible that the build‐up of these pathological proteins is at least partially due to reduced glymphatic clearance. Accordingly, it has been shown that just 6 h of acute sleep deprivation increases levels of Aβ and tau by up to 50% in mice and humans. Recovery sleep brings these levels back down (Kang et al. 2009; Holth et al. 2019; Hauglund et al. 2020; Lucey et al. 2019). In PD, sleep deprivation and increased neuronal activity lead to the build‐up of pathological αSyn, while SWS induced by sodium oxybate reduces αSyn accumulation (Morawska et al. 2021; Yamada and Iwatsubo 2018).

Perhaps the most striking evidence for the role of sleep disruptions in neurodegeneration is the statistic that 80% of patients with rapid eye movement sleep behaviour disorder (a disorder in which patients lose muscle atonia and act out their dreams) develop Parkinson's or Lewy Body Dementia within 10 years (Hu 2020). Thus, the chronic sleep disruptions that are characteristic of AD and PD could directly contribute to neurodegeneration by increasing the build‐up of pathological proteins.

As SCRDs are such a ubiquitous and disruptive symptom in neurodegenerative disorders, many patients are prescribed sleeping aids or sedatives. These range from bright light therapy, melatonin receptor agonists and sedative antidepressants to BZDs, Z‐drugs and DORAs (McCleery and Sharpley 2020; Voysey et al. 2021). While many of these have been confidently prescribed to treat insomnia and other comorbidities, few studies have examined how these agents contribute to the pathology of the particular disorder they are treating. Evidence suggests this is a crucial aspect of sleep medicine that cannot be ignored.

Low doses of antidepressants such as selective serotonin reuptake inhibitors or trazodone have been used frequently to treat insomnia in individuals with neurodegenerative diseases, but there are few data to support their use, and these drugs produce the side effects of daytime sleepiness, dizziness and weight gain—leading to obstructive sleep apnoea (Mendelson 2005). Melatonin has also been used to try and improve sleep in dementia, but the effects are either small or absent (Wennberg et al. 2017). As previously mentioned, antihistamines are widely found in many over‐the‐counter sleep aids (e.g., diphenhydramine‐based antihistamine Benadryl), but should be avoided in Alzheimer's because they can reduce acetylcholine in the brain, which makes the cognitive impairment worse (Molano and Vaughn 2014). In fact, a study on men and women aged 65 or older found that people who used diphenhydramine‐based drugs were more likely to develop dementia, and the dementia risk increased with extended use. Incredibly, taking diphenhydramine for 3 years or more was associated with a 54% higher dementia risk than taking the same dose for 3 months or less (Gray et al. 2015).

BZD and Z‐drug are prescribed sedatives that have been shown to reduce SWS in healthy individuals (Arbon et al. 2015). Given the fundamental role of SWS in clearing waste from the brain, it is perhaps unsurprising that cognitive decline is markedly worse in patients treated with BZDs (Ellul et al. 2007). Indeed, midazolam (a BZD) has been shown to directly increase brain tau phosphorylation in WT and AD model mice (Whittington et al. 2019). Thus, BZDs, which can be prescribed to help treat symptoms of neurodegeneration, could instead be worsening the underlying pathology. It is therefore worth stressing that benzodiazepine‐based drugs are not recommended for dementia or Parkinson's because they can increase the decline in cognition and mood, promote daytime sleepiness and increase the risk of falls. Indeed, a recent review of the use of the newer ‘Z drugs’ (zopiclone, zaleplon, zolpidem, etc.) to treat insomnia in dementia concluded that any benefit was considerably outweighed by the risks (Ettcheto et al. 2019).

As previously mentioned, drugs targeting orexinergic signalling target sleep pathways much more specifically. Orexin itself has also been tied to AD pathology as increased cerebrospinal fluid (CSF) orexin levels are positively correlated to sleep deterioration and CSF tau protein levels in drug‐naïve AD patients (Liguori et al. 2014). Administration of DORA suvorexant has been shown to reduce tau phosphorylation and Aβ concentrations in the CSF of healthy participants (Lucey et al. 2023). Furthermore, infusion of DORA almorexant into the interstitial fluid (ISF) of an amyloidosis mouse model for 24 h suppressed ISF Aβ levels (Kang et al. 2009). Attention to the particular disorder being treated is crucial, however, as there are concerns that suvorexant may promote RBD in Parkinson's patients (Tabata et al. 2017).

Given the crucial role that sleep has in maintaining brain health, we believe that the development of more specific drug therapies for sleep will have widespread benefits for comorbidities, including neurodegenerative disorders. Prescription of sleep medicine must be tailored to the specific disease state of each individual, however, to avoid unintentionally worsening disease progression. In view of the findings to date, current treatment of SCRD in dementia and Parkinson's should carefully consider the harm/benefit ratio of existing sleep medications.

## Author Contributions

**Brooke A. Prakash:** conceptualization, writing – original draft, writing – review and editing, visualization. **Ishani Shah:** writing – original draft, writing – review and editing. **Guohao Ni:** writing – original draft, writing – review and editing. **Aarti Jagannath:** conceptualization, writing – original draft, writing – review and editing. **Russell G. Foster:** conceptualization, writing – original draft, writing – review and editing. **Sridhar Vasudevan:** conceptualization.

## Conflicts of Interest

Aarti Jagannath, Sridhar Vasudevan and Russell G. Foster are founders and shareholders of Circadian Therapeutics; however, this does not influence the work presented in this paper. All other authors declare that they have no conflicts of interest.

## Data Availability

Data sharing is not applicable to this article as no new data were created or analyzed in this study.
